# Changes of intraocular pressure in different trimesters of pregnancy among Syrian refugees in Turkey: A cross-sectional study

**DOI:** 10.4274/tjod.40221

**Published:** 2016-06-15

**Authors:** Harun Egemen Tolunay, Sait Coşkun Özcan, Yavuz Emre Şükür, Deniz Özarslan Özcan, Fatih Mehmet Adıbelli, Neşe Gül Hilali

**Affiliations:** 1 Suruç State Hospital, Clinic of Obstetrics and Gynecology, Şanlıurfa, Turkey; 2 Keçiören Training and Research Hospital, Clinic of Obstetrics and Gynecology, Ankara, Turkey; 3 Akçakale State Hospital, Clinic of Ophthalmology, Şanlıurfa, Turkey; 4 Harran University Faculty of Medicine, Department of Obstetrics and Gynecology, Şanlıurfa, Turkey

**Keywords:** Pregnancy, intraocular pressure, Syrian refugees

## Abstract

**Objective:**

To evaluate the physiologic changes in intraocular pressure associated with pregnancy in healthy Syrian refugee women in Turkey.

**Materials and Methods:**

In this cross-sectional study, intraocular pressures were measured using a Goldmann tonometer in 235 patients in the first, second, and third trimester of pregnancy and puerperium among Syrian refugees in Turkey.

**Results:**

Mean intraocular pressures values of the right eye were 15.5±2.5 mmHg, 14.4±1.4 mmHg, 13.9±1.6 and 14.7±1.9 mmHg in the three trimesters and puerperium, respectively. Mean intraocular pressures values of the left eye were 15.3±1.6 mmHg, 14.3±1.4 mmHg, 13.9±1.6 and 15.3±2.2 mmHg in the three trimesters and puerperium, respectively. The mean intraocular pressures values measured from both eyes were significantly higher in first trimester and puerperal period than in the third trimester (p<0.001).

**Conclusion:**

Changes in the intraocular pressure in pregnancy are common and temporary. This study shows the baseline changes in the intraocular pressure during pregnancy in healthy women. Therefore, we cannot extrapolate the results to the whole eye. A decrease in intraocular pressures was shown in healthy pregnant women.

## INTRODUCTION

Pregnancy is a complex physiologic process that affects all organic systems. Ocular changes in pregnancy can be physiologic or pathologic. Decrease in intraocular pressure (IOP) in pregnancy has been shown in the literature^(1)^. Most physiologic changes due to pregnancy are usually marked in last trimester, in which the hormonal status is at its peak^(2)^. In pregnancy the probable reasons of the reduction of the IOP are the increase in the outflow of aqueous humor (AH), physiologic relaxation of ligaments and reduction of cornea rigidity that occurs in late pregnancy, the reducing effect of diminished venous pressure in upper extremities on episcleral venous pressure, vasodilator effects of pregnancy hormones, and pregnancy acidosis^(3)^.

Due to hormonal influences, physiologic ocular changes have been shown in pregnancy in Caucasian women. Cornea sensitivity, refractive status, IOP, and visual acuity can change in pregnancy^(4,5,6)^. Therefore, it is important to be aware of physiologic changes as well as of the potential effects on preexisting disease and complications in order to counsel and advise women who are currently pregnant. The aim of our research was to observe the physiologic varieties of IOP in pregnant Syrian refugees in Turkey.

## MATERIALS AND METHODS

A cross-sectional study was performed after obtaining approval from the Ethics Commission of the Harran University Faculty of Medicine, Şanlıurfa, Turkey. All patients provided written informed consent. Patients were selected in the obstetrics and gynecology outpatients clinic of Suruç State Hospital and Harran University Faculty of Medicine between August 5^th^, 2015, and September 13^th^, 2015. Two hundred-eighty pregnant women were evaluated for suitability during the research period. Eighteen patients did not meet the inclusion criteria and 23 patients refused to participate in the study. Four patients were excluded from the study during IOP measurement because of excessive blinking. In total, 235 patients were included in the research. The inclusion criteria were to be a female Syrian refugee aged between 20-35 years with a known last menstrual period. The exclusion criteria were the presence of any systemic disease such as hypertensive disorders, diabetes mellitus, or any ocular disease. Patients with preeclampsia and gestational diabetes were treated and managed in accordance with current guidelines, but these patients were not evaluated in the research. In addition, we did not include any twin pregnancies in the study. The study groups consisted of pregnant women in the first, second, and third trimesters, and women with puerpera. The first third or 14 weeks of pregnancy were defined as the first trimester, 14-28 weeks of pregnancy were defined as the second trimester, and the last third of pregnancy was defined as the third trimester. Puerperium is defined as the period from the end of labor until involution of the uterus is complete, usually lasting between 3 and 6 weeks. Patients were assessed in clinical and ultrasonographic examinations during antenatal screening. Each patient’s age, parity, and smoking status was recorded. The smoking rate was low in our patient population. Patients who smoked more than 5 cigarettes a day were not included in the study.

A full ophthalmoscopic examination was performed to exclude any anterior and posterior segment illness. The IOPs were evaluated with the same Goldmann tonometer (Optilasa, S.L., Madrid, Spain). The device was calibrated prior to the study. One drop of 0.5% proparacain was instilled into the each eye of the subjects and both inferior conjunctival sacs were touched with a dry fluorescein strip (Biotech, Gujarat, India) to measure the IOP of the eyes; as soon as a value was established it was recorded. The right eye was always measured first. All measurements were performed in the morning between 08:00 AM and 10:00 AM to avoid the diurnal variation of IOP.

### Statistical Analyses

The statistical package for the social sciences (SPSS) version 20.0 for Windows was used for all statistical analyses. The Shapiro-Wilk test was used to test distribution of normality. According to the results, parametric tests were preferred. We used one-way ANOVA test to compare continuous variables. Categorical variables were compared with the chi-square test. A p value <0.05 was considered statistically significant. When we found a statistically significant difference, we performed a post-hoc analysis between all group pairs to determine the source of statistical significance.

## RESULTS

There were 61 (25.9%) patients in the 1^st^ trimester, 76 (32.3%) patients in the 2^nd^ trimester, 54 (22.9%) patients in the 3^rd^ trimester, and 44 (18.7%) patients in the puerperal period. The average age of the patients was 27.4±4.7 years. We found no statistically significant difference in age between the groups (p=0.167). The mean parity number of the patients was 3.2±0.2. Similarly, the mean parity number and ratio of smokers within the groups were also comparable (p=0.310, p=0.052, respectively) (Table 1).

Table 2 summarizes the mean IOP values of the studied population. The mean IOP values measured from the right eye were significantly higher in the first trimester and puerperal period than in the third trimester (15.5±2.5 mmHg, and 14.7±1.9 mmHg vs. 13.9±1.6 mmHg, respectively; p<0.001). The mean IOP values measured from the left eye were significantly higher in the first trimester and puerperal period than in the second and third trimesters (15.3±1.6, and 15.3±2.2 mmHg, vs. 14.3±1.4 mmHg and 13.9±1.6 mmHg, respectively (p<0.001).

## DISCUSSION

This is the first study, to our knowledge, to examine the physiologic changes of IOP in pregnant Syrian refugees in Turkey. The present cross-sectional research was conducted to evaluate the relationship between pregnancy period and IOP. Our results clearly demonstrate that the IOP values decrease as the gestational period progresses and return to normal in the puerperal period. The lowest IOP values were detected in the third trimester of pregnancy. Early studies revealed the effects of pregnancy on the eyes, which in addition to new changes and pre-existing ocular disorders, may change their course owing to the widespread changes during pregnancy, hormonal and otherwise, that may either be exacerbated or ameliorated^(7)^.

The changes in IOP during pregnancy were significant in our study. There was a decline in IOP from the first trimester to the third trimester. These changes are frequently temporary and returned to normal levels after delivery^(8)^. Our finding of an ocular hypotensive effect in the third trimester of pregnancy is consistent with other studies in the literature^(1,3,9,10)^. Otherwise, Philips and Gore^(11)^ reported no significant difference in the ocular hypotensive effect of late pregnancy in women who were normotensive and hypertensive.

The main physiological mechanism responsible for the decrease in IOP during pregnancy is not fully known. The decrease in IOP during pregnancy is likely multifactorial^(12)^. The decreased IOP in pregnancy may be due to elevated hormonal levels, which cause an increase in fluid outflow conductance without altering the rate of fluid entry^(13)^. It is well documented that progesterone, estrogen, and other placental hormone levels change during pregnancy. Estrogen has a dilatator effect on the vessels. Omoti et al.^(10)^ reported that this vasodilator effect provides a decrease of arterial pressure and thus causes a reduction of AH production.

In pregnancy there is a general decrease in peripheral vascular resistance. Therefore, episcleral venous pressure also decreases in pregnancy^(14,15)^. AH outflow is facilitated by this decrease. Also estrogen has a protective effect in the vascular pathology by the production of mediators such as nitric oxide, prostacyclins, endothelin-I, and eicosanoids. These vasodilators cause reduction of resistance^(8)^. In pregnancy with a normal production of AH, the facility of AH drainage is due to the increased levels of the β-hCG and progesterone, and general decreased peripheral vascular resistance. This results in a gradual, statistically significant decrease in IOP during pregnancy^(1)^. The anti-glucocorticoid features of progesterone may have a role in the reduction of IOP. Endogen corticosteroids have an ocular hypertensive effect and progesterone blocks this effect^(16)^. During pregnancy, relaxin is released by the high levels of estrogen. Relaxin is a hormone that has softening properties and in late pregnancy these elastic changes decrease cornea-scleral rigidity and this causes a decrease in IOP by the diminished production of AH^(11)^. The effect of relaxin on outflow facility is thought to be mediated by collagen changes, which in turn affect the rigidity of Schlemm’s canal and the trabecular meshwork^(17)^. Saylık and Saylık^(18)^ reported that the reduction of IOP was more pronounced in twin pregnancies than in singleton pregnancies because of the presence of higher levels of hormones that affect IOP in twin pregnancies. Qureshi et al.^(19)^ showed that the ocular hypotensive effect of late pregnancy was significantly greater in multigravida women than in those who were primigravida. In our study, the average parity number was 3.2±0.2. The multigravida nature of our patients might have increased the relaxin hormone levels, which could explain the diminished IOP in pregnancy in this research.

The small sample size and the cross-sectional study design were the major limitations of the present study, which prevent drawing definitive conclusions about the progression of IOP. A longitudinal design is necessary to establish the change of IOP in different trimesters of pregnancy. However, we were unable to collect data from a cohort during all pregnancy periods due to the inconsistency of Syrian pregnant women in attending ante-natal follow-up programs in this region of the country.

## CONCLUSION

Changes in the IOP in pregnancy are common and temporary. However, these may have an impact on the progression of a preexisting ocular disease. Physicians should know about these physiologic changes in pregnancy so as not to consider these changes pathologic. At the same time, the physiologic decrease in IOP could be advantageous for pre-existing glaucoma. Therefore, physiologic changes should be kept in mind in order to prevent misdiagnoses during routine antenatal investigations.

## Figures and Tables

**Table 1:**
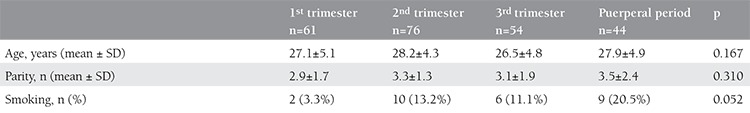
Demographics of the study groups

**Table 2:**
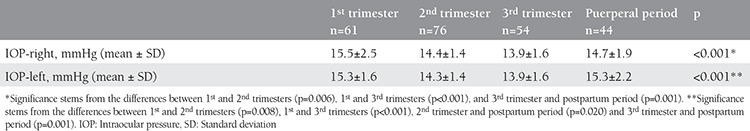
Intraocular pressure values of the study groups
